# Association between dental plaque index and COVID-19 severity: a cross-sectional study in a conflict-affected humanitarian setting

**DOI:** 10.1186/s12903-026-09169-7

**Published:** 2026-07-04

**Authors:** Hussam Ismail, Fouad Al-Daoud, Gohar Mushtaq

**Affiliations:** 1 Faculty of Dentistry, Idlib University, Idlib, Syria; 2 Department of Laboratory Diagnosis, Faculty of Medicine, Idlib University, Idlib, Syria

**Keywords:** COVID-19, SARS-CoV-2, Dental plaque, Oral hygiene, Oral microbiome, Respiratory diseases, Cross-sectional study, Humanitarian crisis, Conflict-affected settings, Northwest Syria

## Abstract

**Background:**

COVID-19 shows marked variation in clinical severity. Identifying demographic and clinical factors associated with severity is particularly important in conflict-affected, resource-limited settings. This study assessed the relationship between COVID-19 symptom severity and selected variables, including sex, age, marital status, and dental plaque index, among patients in northwest Syria.

**Methods:**

This cross-sectional analytical study was conducted from 12 October to 23 November 2021 in three COVID-19 isolation centers in northwest Syria. Sixty adult patients with confirmed SARS-CoV-2 infection were enrolled through consecutive screening with purposive quota balancing by clinical severity category, resulting in 20 patients in each of the mild, moderate, and severe groups. Demographic data were recorded, and oral examination was performed by one trained examiner to assess plaque index using the modified Greene–Vermillion index. Associations were analyzed using Spearman’s rank correlation, Pearson correlation, Kruskal–Wallis H test, and chi-squared test, with statistical significance set at *p* ≤ 0.05.

**Results:**

The sample included 38 females (63.3%) and 22 males (36.7%), aged 18–82 years. COVID-19 severity was significantly associated with sex, with greater severity among males (Spearman’s ρ = − 0.428, *p* = 0.001). Age showed a weak but significant positive correlation with severity (Pearson’s *r* = 0.287, *p* = 0.026). Marital status was also associated with severity (Spearman’s ρ = 0.329, *p* = 0.010), although this relationship appeared to be strongly confounded by age. Dental plaque index showed a moderate positive association with COVID-19 severity (Spearman’s ρ = 0.533, 95% CI: 0.307–0.702; *p* < 0.001), indicating that higher plaque accumulation was associated with more severe symptoms.

**Conclusion:**

In this conflict-affected humanitarian setting, male sex, older age, and higher dental plaque index were significantly associated with increased COVID-19 symptom severity. These findings are preliminary and should be interpreted cautiously because of the cross-sectional design, small sample size, and limited data on potential confounders. Nevertheless, the study contributes evidence from an underrepresented crisis-affected population and suggests that oral health assessment may have value as part of broader COVID-19 risk evaluation in resource-constrained settings.

## Introduction

The oral cavity contains a complex and highly dynamic microbial ecosystem that plays a fundamental role in maintaining the balance between oral and systemic health [1]. This ecosystem is made up of bacteria, archaea, fungi, protozoa, and viruses that coexist within organized communities and constantly interact with each other and the host immune system [2, 3]. In physiological conditions, this microbial community contributes to internal homeostasis; however, the disruption of this balance - oral dysbiosis - is associated with a wide spectrum of inflammatory and systemic disorders.

Dental plaque represents an organized polymicrobial biofilm that adheres to the tooth surface and is embedded within an extracellular polymeric matrix consisting mainly of microbial products and host-derived components [4]. It is organized as a highly coordinated microbial community and not as a random accumulation of microorganisms. The biofilm adheres to the acquired enamel layer, a saliva-derived protein layer composed mainly of glycoproteins and other large salivary molecules [5, 6]. The accumulation and maturation of dental plaque promote inflammatory responses in adjacent gingival tissues and may contribute to the burden of systemic inflammation.

Coronavirus disease 2019 (COVID-19), caused by severe acute respiratory syndrome coronavirus 2 (SARS-CoV-2), was first identified in Wuhan, China, in late 2019 and has rapidly developed into a global pandemic, leaving significant health, social and economic impacts around the world [7, 8]. COVID-19 exhibits a broad clinical spectrum, with many reported manifestations ranging from asymptomatic infection to severe respiratory failure and multiple organ dysfunction [9]. Although the SARS-CoV-2 virus primarily targets the respiratory system, the involvement of organs outside the lung—including the kidneys, cardiovascular system, and liver—has been extensively documented [10]. Deaths are mainly associated with severe respiratory complications, including viral pneumonia, acute respiratory distress syndrome (ARDS), and systemic inflammatory responses [11].

Due to the anatomical and functional continuity between the oral cavity and the respiratory tract, oral health status may influence susceptibility to respiratory infections through aspiration of oral pathogens, modulation of mucosal immunity, and amplification of systemic inflammatory pathways [9, 12, 13]. This concept is supported by the “mouth–lung axis,” in which dental plaque and oral dysbiosis may serve as reservoirs for opportunistic respiratory pathogens and may contribute to inflammatory burden in susceptible individuals [14–17]. Poor oral hygiene, gingivitis, and microbial biofilm accumulation have also been linked to respiratory disease risk and may theoretically exacerbate systemic inflammation in severe COVID-19 [18–21].

Research on oral–systemic health interactions in humanitarian and conflict-affected settings remains limited. These contexts are characterized by disrupted healthcare infrastructure, limited diagnostic and therapeutic resources, and populations with deteriorating health conditions due to malnutrition, displacement, and trauma. Understanding whether simple clinical indicators, such as plaque accumulation, are associated with infectious disease severity may help guide practical, low-cost assessment strategies in resource-constrained settings.

In this study, conducted in northwest Syria during an active humanitarian crisis, dental plaque was selected as a measurable clinical indicator of oral hygiene status to investigate its association with COVID-19 severity. In addition, demographic variables, including age and sex, were examined to assess their relationship with disease severity in this underrepresented population. We emphasize that this cross-sectional design precludes causal inference, and all observed relationships should be interpreted as correlations requiring confirmation by future longitudinal investigations in more stable healthcare settings.

## Materials and methods

### Study design and environment

This study was designed as a cross-sectional analysis conducted between 12 October 2021 and 23 November 2021. The study population included patients admitted to three dedicated COVID-19 isolation centers in northwest Syria: Violet Center (Jericho City), Emergency Response Center (Idlib City), and Intensive Care Center (Idlib City).

Northwest Syria has experienced ongoing armed conflict, resulting in a severe deterioration in healthcare infrastructure, the displacement of health professionals, and the intermittent disruption of essential medical supplies. During the COVID-19 pandemic, these prior gaps were exacerbated by limited testing capacity, scarcity of personal protective equipment, and high patient burden. The three designated centers represented the basic health facilities available for the diagnosis and management of SARS-CoV-2 in the region during the study period.

We recognize that conducting research in an active conflict zone poses unique ethical and logistical challenges. The security situation prevented random sampling, longitudinal follow-up, or access to advanced diagnostic tools. However, we believe that generating evidence under these restrictive conditions serves an important public health function for populations that are often underrepresented in biomedical literature.

### Study population and sampling

Eligible patients admitted to the three designated COVID-19 isolation centers were consecutively screened during the six-week study period. To ensure balanced group sizes for meaningful statistical comparison, enrollment was monitored by clinical severity category, with recruitment continuing until 20 patients were included in each of the mild, moderate, and severe groups. This approach represents consecutive screening with purposive quota balancing by severity stratum. The final sample comprised 60 adult patients aged ≥ 18 years with permanent teeth. No formal a priori sample-size calculation was performed; the sample size was determined by the number of eligible patients available during the study period, reflecting the limited patient flow and operational constraints in this conflict-affected, resource-limited context. Therefore, the findings should be interpreted as preliminary and should be confirmed in larger multicenter studies.

Patients were divided into three groups according to the clinical severity of COVID-19, with 20 patients assigned to each group:

#### Group I (mild disease)

Patients with mild symptoms, such as fever, cough, fatigue, myalgia, or sore throat, without dyspnea or clinical/radiological evidence of pneumonia, and who did not require oxygen supplementation. SpO₂ was ≥ 94% on room air.

#### Group II (moderate disease)

Patients with clinical signs of pneumonia, such as fever, cough, tachypnea, or respiratory rate > 20 breaths/minute, requiring hospitalization and supplemental oxygen by nasal cannula or face mask to maintain SpO₂ ≥90%. Radiological evidence of pulmonary infiltration was recorded when imaging was available.

#### Group III (severe disease)

Patients with severe respiratory distress, respiratory rate > 30 breaths/minute, SpO₂ <90% on room air or despite supplemental oxygen, or those requiring high-flow nasal oxygen, non-invasive ventilation, invasive mechanical ventilation, or ICU admission for hemodynamic support or organ failure management.

SARS-CoV-2 infection was confirmed using a reverse transcriptase polymerase chain reaction (RT-PCR) test conducted at the respective isolation centers using available laboratory capacity. The disease severity was classified according to the WHO’s interim guidelines for the management of COVID-19 [22]. We acknowledge that the availability of radiography, arterial blood gas analysis, and advanced monitoring has been inconsistent across centers due to limited resources, and the severity classification has been based primarily on clinical assessment and pulse oximetry.

The criteria for inclusion were: (1) confirmation of SARS-CoV-2 infection by RT-PCR; (2) age ≥ 18 years; (3) permanent teeth; and (4) willingness to participate and provide informed consent. The exclusion criteria were: (1) edentulous patients or patients without sufficient permanent teeth for plaque assessment; (2) patients with acute oral infections or dental abscesses at the time of examination; (3) patients with known bleeding disorders that prevent oral examination; and (4) patients unable to cooperate with the oral examination due to severe respiratory distress or a change in mental state.

### Oral examination and plaque evaluation

Oral examinations were performed by one trained examiner (a licensed periodontologist with ten years of clinical experience) using an oral mirror, William probe, and head-mounted lighting to ensure optimal intraoral vision. The examiner underwent calibration training using standardized photographic reference images prior to data collection. The use of a single examiner was a practical decision, reflecting the scarcity of dental professionals available in the area during the study period. Formal intra-examiner reliability testing was not performed because of operational constraints during the COVID-19 emergency. To reduce measurement variability, all examinations were performed by a single trained periodontologist using standardized criteria and photographic reference images before data collection.

Dental plaque index was assessed using the modified Greene-Vermillion Index (Simplified Oral Hygiene Index – OHI-S Component) [23, 24]. This index was chosen because it requires little equipment, can be performed quickly at the bedside, and does not require specialized periodontal disease devices that were not available in isolation centers. The teeth were divided into six sextants, three in the maxillary arch and three in the mandibular arch. Plaque scores were recorded on both the facial and lingual or palatal surfaces of teeth within each sextant (12 surfaces of each patient).

Plaque scores were assigned according to the following criteria:

- Score 0: No visible plaque on the surface of the tooth.
- Score 1: Plaque covers less than one-third of the tooth’s surface.
- Score 2: A plaque covering more than one-third of the tooth’s surface but less than two-thirds of the tooth’s surface.
- Score 3: Plaque covers more than two-thirds of the tooth surface.

The total plaque index score of each patient was calculated based on the arithmetic mean of the twelve recorded surface values. For descriptive presentation in Table 4, these continuous mean scores were rounded to the nearest integer (0, 1, 2, or 3) to align with the original ordinal scoring criteria and to facilitate clinical interpretability. All inferential statistical analyses were performed using the continuous (unrounded) mean plaque index values. Oral examinations were performed during active hospitalization or isolation and were generally completed within 48 h of admission. The timing of oral examination in relation to symptom onset was documented when available. However, the exact timing in relation to oxygen therapy, non-invasive ventilation, ICU-level care, or other intensive supportive treatment was not consistently recorded. This was therefore considered a potential source of reverse causality and was addressed in the Discussion and Limitations sections.

Infection control protocols followed standard precautions as much as possible due to resource constraints, including the use of personal protective equipment (surgical mask, face shield, gloves, gown), disinfection of surfaces, and the use of disposable instruments. The examiner was vaccinated against COVID-19 and underwent regular health monitoring during the study period. Disposable instruments were discarded immediately after examination, and reusable equipment, including the head-mounted light, was disinfected between patients according to locally available infection-control procedures. The plaque index was used as a simple bedside indicator of oral hygiene status, not as a comprehensive measure of periodontal inflammatory burden.

The modified Greene and Vermillion plaque index (Simplified Oral Hygiene Index — OHI-S plaque component) was selected as the primary oral health indicator because it is rapid, requires minimal bedside equipment, avoids prolonged periodontal examination in COVID-19 isolation settings, and reflects visible microbial biofilm accumulation. However, this index was used only as a practical indicator of oral hygiene status and does not capture gingival inflammation, bleeding on probing, periodontal pocket depth, or clinical attachment loss.

### Statistical analysis

Data were entered and analyzed using IBM SPSS Statistics for Windows, version 25.0 (IBM Corporation, Armonk, New York, USA). Categorical variables were summarized using frequencies and percentages. Continuous variables were expressed as mean ± standard deviation (SD) or median (interquartile range [IQR]) depending on the distribution evaluated by the Shapiro-Wilk test.

Since the severity of COVID-19 is an ordinal variable with three ordered categories (mild, moderate, and severe) and the plaque index is a continuous variable derived from the mean of twelve ordinal surface scores (0–3), we used the following analytical approach:

Spearman’s rank correlation coefficient (ρ) was used as the primary analysis to evaluate monotonic associations between COVID-19 severity and the main study variables, including the unrounded mean plaque index values, sex, and marital status.

The Pearson correlation coefficient (r) was used as a secondary analysis for continuous variables, particularly age, when linearity and distributional assumptions were considered acceptable. We acknowledge that these assumptions may not be fully satisfied when analyses involve ordinal severity categories.

The Kruskal–Wallis H test was used to compare the distribution of plaque indices across the three severity groups, followed by the Mann–Whitney pairwise tests with Bonferroni’s correction for subsequent comparisons.

Chi-square tests (χ²) or Fisher’s exact tests were used to examine associations between categorical variables (sex, marital status) and severity groups.

Correlation coefficients (r or ρ) with 95% confidence intervals (CIs) were calculated. A two-tailed p-value < 0.05 was considered statistically significant. No post hoc power analysis was used to support the interpretation of the findings. Instead, the strength and precision of the observed associations were evaluated using effect sizes, p-values, and 95% confidence intervals. Because of the small sample size, the results should be interpreted cautiously and considered exploratory.

Multivariable ordinal logistic regression was considered but not performed because of the small sample size and the absence of systematic data on key confounders, including smoking, comorbidities, vaccination status, socioeconomic status, periodontal status, and oral hygiene behavior. Therefore, the analyses were limited to exploratory univariate associations, and the results should be interpreted cautiously.

## Results

### Distribution by sex

The total sample consisted of 38 females (63.3%) and 22 males (36.7%). Distribution by disease severity showed significant sex-related disparity (Table 1). Mild manifestations were observed mainly among females (17 females vs. 3 males), while severe manifestations were more common among males (13 males vs. 7 females). Chi-square analysis revealed a statistically significant association between sex and the severity groups (χ² = 14.52, *p* = 0.001).

**Table 1:** Distribution of the study sample by sex across COVID-19 severity groups

Severity Group	Male, *n* (%)	Female, *n* (%)	Total, *n* (%)
Mild	3 (15.0)	17 (85.0)	20 (100)
Moderate	6 (30.0)	14 (70.0)	20 (100)
Severe	13 (65.0)	7 (35.0)	20 (100)

χ² = 14.52, *p* = 0.001

### Age distribution

Patients ranged in age from 18 to 82 years in the specific severity groups. Mean age gradually increased with symptom severity: mild symptoms: mean age 47.5 ± 12.3 years; moderate symptoms: mean age 52.0 ± 14.1 years; severe symptoms: mean age 52.5 ± 13.8 years. Although the mean age differences were modest, statistical analysis showed a weak but statistically significant positive relationship between age and severity of COVID-19 symptoms (Pearson *r* = 0.287; 95% CI: 0.031–0.508; *p* = 0.026), suggesting that increased age was associated with increased disease severity. The Kruskal–Wallis test did not reveal statistically significant differences in age distributions across severity groups (H = 1.42, *p* = 0.49). The age distribution across COVID-19 severity groups is shown in Table 2.

**Table 2:** Age distribution across COVID-19 severity groups

Severity Group	*n*	Mean ± SD (years)	Median (IQR)	Range (years)
Mild	20	47.5 ± 12.3	46 (38–56)	18–70
Moderate	20	52.0 ± 14.1	51 (42–61)	20–85
Severe	20	52.5 ± 13.8	53 (43–62)	28–82

r Pearson = 0.287, 95% CI: 0.031–0.508, *p* = 0.026. Kruskal–Wallis H = 1.42, *p* = 0.49

### Distribution of marital status

Of the total sample, 50 patients (83.3%) were married, and 10 patients (16.7%) were single. Distribution across severity groups showed a higher frequency of married individuals in the moderate and severe groups: mild symptoms: 13 married (65.0%), 7 single (35.0%); Moderate symptoms: 18 married (90.0%), 2 single (10.0%); Severe symptoms: 19 married (95.0%), 1 single (5.0%).

A statistically significant positive relationship was identified between marital status and severity of COVID-19 symptoms (Spearman ρ = 0.329; 95% CI: 0.075–0.543; *p* = 0.010). Furthermore, a strong positive association was observed between marital status and patient age (ρ Spearman = 0.62; 95% CI: 0.415–0.765; < 0.001), suggesting that age may be a confounding factor influencing the relationship between marital status and condition severity. Chi-square analysis confirmed a significant association between marital status and the severity group (χ² = 6.67, *p* = 0.036). The distribution of marital status across COVID-19 severity groups is presented in Table 3.

**Table 3:** Distribution of marital status across COVID-19 severity groups

Severity Group	Married, *n* (%)	Single, *n* (%)	Total, *n* (%)
Mild	13 (65.0)	7 (35.0)	20 (100)
Moderate	18 (90.0)	2 (10.0)	20 (100)
Severe	19 (95.0)	1 (5.0)	20 (100)

Spearman ρ = 0.329, 95% CI: 0.075–0.543, *p* = 0.010; χ² = 6.67, *p* = 0.036

### Distribution of the plaque index

Clinical oral examination revealed plaque index scores ranging from 0 to 3 across all severity categories. In the mild group, the plaque index values were mostly 1 and 2 (score 0: *n* = 2; score 1: *n* = 9; score 2: *n* = 9; score 3: *n* = 0). In the moderate group, most patients showed a plaque index of 2 (score 0: *n* = 1; score 1: *n* = 2; score 2: *n* = 16; score 3: *n* = 1). In the severe group, the highest plaque index scores (2 and 3) were predominant, with 7 patients showing a score of 3 (score 0: *n* = 1; score 1: *n* = 0; score 2: *n* = 12; score 3: *n* = 7).

The Kruskal–Wallis test revealed statistically significant differences in the distribution of plaque indices across the severity groups (H = 18.72, *p* < 0.001). Pairwise comparisons with Bonferroni’s correction showed significant differences between mild versus severe (*p* < 0.001) and moderate versus severe (*p* = 0.012) groups, but not between mild versus moderate (*p* = 0.18) groups.

Statistical analyses showed a statistically significant moderate positive association between dental plaque index and the severity of COVID-19 symptoms (Spearman ρ = 0.533; 95% confidence interval: 0.307–0.702; *p* < 0.001). This finding suggests that increased plaque accumulation was associated with increased severity of SARS-CoV-2 infection. However, given the cross-sectional design and the humanitarian context in which the data were collected, where severe illness may have profoundly affected patients’ ability to maintain oral hygiene, the temporal trend of this association cannot be determined. Reverse causation remains a plausible alternative explanation. The distribution of plaque index scores across COVID-19 severity groups is shown in Table 4.

**Table 4:** Distribution of plaque index scores across COVID-19 severity groups

Plaque Index	Mild, *n* (%)	Moderate, *n* (%)	Severe, *n* (%)
0	2 (10.0)	1 (5.0)	1 (5.0)
1	9 (45.0)	2 (10.0)	0 (0.0)
2	9 (45.0)	16 (80.0)	12 (60.0)
3	0 (0.0)	1 (5.0)	7 (35.0)

Plaque index categories represent rounded patient-level mean plaque index values derived from twelve surface scores. All inferential analyses were performed using the continuous unrounded mean plaque index values. Kruskal–Wallis H = 18.72, *p* < 0.001. Spearman ρ = 0.533, 95% confidence interval: 0.307–0.702, *p* < 0.001

### Correlation analysis summary

The associations between COVID-19 severity and the study variables are summarized in Table 5.

**Table 5:** Summary of the analysis of the association between the severity of COVID-19 and the study variables

Variable Pair	Testing Used	Coefficient	95% CI	*p*-value	Interpretation
Severity vs. Sex	Spearman	ρ = − 0.428	–0.622 to –0.184	0.001	Male sex associated with increased severity
Severity vs. Age	Pearson	*r* = 0.287	0.031 to 0.508	0.026	Weak positive association
Severity vs. Marital Status	Spearman	ρ = 0.329	0.075 to 0.543	0.010	Modest association; likely confounded by age
Severity vs. Plaque Index	Spearman	ρ = 0.533	0.307 to 0.702	< 0.001	Moderate positive correlation

All p-values have two tails. CI = Confidence Interval. 95% CI for correlation coefficients indicates the precision of estimates; narrower intervals indicate more precise estimates. The mean correlation between plaque index and severity of COVID-19 (ρ = 0.533) indicates a meaningful correlation, although the wide interval (0.307–0.702) reflects uncertainty due to the small sample size. The direction of this association cannot be determined from cross-sectional data collected during active disease

## Discussion

The current study showed several statistically significant associations between the severity of COVID-19 symptoms and the demographic and clinical variables examined among patients in northwest Syria during an active humanitarian crisis. These findings are consistent with previously published data from more stable healthcare settings and further support the multifactorial nature of COVID-19 severity. However, we emphasize that this cross-sectional design, small sample size, and highly constrained humanitarian context preclude causal inference, and all observed relationships should be interpreted as preliminary associations requiring confirmation through future longitudinal investigations in more appropriate research settings. Importantly, the study was not powered by a formal sample-size calculation, and the small sample size limits the precision of estimates, the ability to detect weaker associations, and the generalizability of the findings.

### Relationship between sex and severity of SARS-CoV-2 infection

The results revealed a statistically significant relationship between the patient’s sex and the severity of symptoms, suggesting that male patients were more severely affected than female patients. This observation is consistent with previously published evidence from diverse geographic settings showing that males are at greater risk of severe clinical outcomes after SARS-CoV-2 infection [25].

A study conducted by Jin et al. in China concluded that male sex is an independent risk factor for worse outcomes and mortality among COVID-19 patients, regardless of age and underlying comorbidities [26]. These findings confirm current findings and reinforce the sex-based biological vulnerability hypothesis.

Several mechanisms may explain this difference. One proposed explanation relates to differential expression of the ACE2 receptor, which serves as a primary cellular entry point for the SARS-CoV-2 virus. Increased expression of ACE2 in males may promote virus entry and replication, increasing susceptibility to severe infection [27]. In addition, immune differences between the sexes may contribute significantly. It has been well established that females generally show stronger innate and adaptive immune responses to viral infections, possibly due to hormonal modulation and X-linked immune-related genes [28]. This improved immune response may give partial protection against the progression of severe disease in women.

In the context of northwest Syria, lifestyle factors may contribute to this disparity. Higher rates of tobacco use and limited access to preventive health care among males in the region have been documented in human health assessments, and these factors may be associated with increased lung impairment and systemic inflammation, which may increase the severity of COVID-19 [29]. However, we were not able to systematically collect smoking or occupational exposure status data in the current study due to time constraints and the absence of standardized reception questionnaires in isolation centers.

Overall, these biological and behavioral factors may partly explain the greater severity observed among male patients in this study. However, we acknowledge that smoking status and other lifestyle factors were not measured in our study, and their potential confounding effect cannot be ruled out.

### The relationship between age and the severity of COVID-19 symptoms

Descriptive and statistical analyses showed a weak but statistically significant positive relationship between the patient’s age and symptom severity. These results suggest that increased age may be associated with a greater likelihood of developing severe manifestations of SARS-CoV-2 infection [30], although the strength of the association in this group was weaker than that observed in the case of sex [31].

These findings are consistent with a comprehensive systematic review by Stark et al., which evaluated 70 studies that looked at the impact of age on COVID-19-related outcomes. Their analysis showed that increasing age was associated with progressively higher risks of hospitalization, severe disease, and adverse outcomes, without identifying a single age threshold at which risk sharply increased [32].

Furthermore, the findings reported by Farshbafnadi et al. from collaborative research conducted between the University of Tehran and the University of Padua (Italy) are consistent with current findings, demonstrating an association between aging and deterioration of clinical outcomes in COVID-19 patients [33].

Pathophysiological causes of age-related disease severity are likely to include immunosenescence, chronic low-grade inflammation (“inflammaging”), endothelial dysfunction, and increased prevalence of comorbid systemic diseases among older adults. In the population of northwest Syria, older adults may also experience cumulative effects of chronic stress, displacement, and disruption of access to chronic disease management, all of which may increase vulnerability to severe COVID-19 due to age.

However, we acknowledge that comorbidity data were not systematically collected in the current study, and the observed association with age and severity may be partly confounded by unmeasured chronic conditions such as diabetes, hypertension, and cardiovascular disease, which are known to increase with age and are proven risk factors for severe COVID-19. The lack of comprehensive medical records in this humanitarian setting has prevented the possibility of reliable verification of comorbidities.

### Correlation between marital status and severity

A modest but statistically significant positive relationship was identified between marital status and symptom severity, with married individuals showing more severe symptoms than single individuals.

This association may be partly explained by socio-environmental and psychological factors. Married people are more likely to live in large households, which increases personal contact and the likelihood of viral exposure or transmission. In addition, married couples may experience greater psychological, social, and financial stress, especially during pandemic-related restrictions, which can negatively affect immune function [34]. In the northwestern Syrian context, married couples – especially males – often have primary responsibility for family security and resource gathering in a conflict environment, which can exacerbate stress-related immune dysregulation.

This explanation is supported by the findings of a study conducted in the United States, which showed that stress and depression associated with financial insecurity during pandemic control measures significantly impacted the health outcomes of middle-class couples [35]. Chronic stress is known to disrupt immune responses and alter the production of cytokines, which can contribute to more severe disease progression.

However, the relationship between marital status and severity must be interpreted with great caution. The strong positive relationship identified between marital status and age in this study (Spearman ρ = 0.62; *p* < 0.001) suggests that age may act as an important confounding variable that influences this association. In many populations, married couples tend to be older than single individuals, and the observed relationship may simply reflect a well-established age-severity relationship rather than an independent influence of marital status. Similar observations were reported in a Turkish study conducted by Kurt, confirming that marital status interacts with several variables, including socioeconomic level, health awareness, and psychological well-being [34].

Therefore, the association between marital status and the severity of COVID-19 requires a broader multivariable investigation to understand the determinants of this association. We caution against interpreting marital status as an independent risk factor based on the current, unadjusted analysis.

### The relationship between plaque index and the severity of COVID-19 symptoms

One of the most notable findings in the current study was the moderately positive relationship between dental plaque index and the severity of COVID-19 symptoms. This relationship suggests that higher plaque index was associated with higher disease severity, although causation cannot be inferred from this cross-sectional design.

Dental plaque is a complex multimicrobial biofilm that contains many pathogenic microorganisms, including respiratory pathogens such as Streptococcus pneumoniae and Haemophilus influenzae [36]. These pathogens may be inhaled into the lower respiratory tract, especially in hospitalized patients or immunocompromised patients, increasing the risk of hospital-acquired pneumonia (HAP). Periodontal disease, caused by chronic plaque buildup, is characterized by persistent inflammation of the tissues that support the teeth. This inflammatory state promotes the systemic diffusion of pro-inflammatory cytokines [37]. The secretion of inflammatory mediators, including interleukin-6 (IL-6) and tumor necrosis factor-α (TNF-α), contributes to systemic immune activation and may contribute to the cytokine storm observed in severe COVID-19 cases [5, 38].

In addition, periodontal disease alters the oral microbiota, facilitating the colonization of pathogenic microorganisms and increasing the systemic inflammatory burden [39, 40]. In COVID-19 patients, who may already have an immune dysregulation, this additional inflammatory load has been hypothesized to be associated with worse clinical outcomes and may increase the likelihood of developing acute respiratory distress syndrome (ARDS).

These findings are consistent with research by Al-Bayaty et al., who reported that dental plaque may act as a reservoir for respiratory pathogens, increasing the susceptibility to hospital-acquired pneumonia and being associated with greater COVID-19 symptom severity through cytokine-mediated mechanisms [36]. Similarly, Gupta et al. have shown associations between oral dysbiosis and the severity of SARS-CoV-2 infection, supporting the biological plausibility of the oral–lung axis [37].

However, we must emphasize the critical limitation of reverse causation. Plaque index in this study was measured after infection with the SARS-CoV-2 virus and during hospitalization or isolation. Therefore, the direction of the correlation is fundamentally unclear. Severe COVID-19 patients, especially those requiring mechanical ventilation or intensive care unit admission, may have a marked reduction in the ability to maintain oral hygiene due to the severity of the disease, sedation, oral dryness, antibiotic use, and limited access to oral care. In the humanitarian context in northwest Syria, where nursing staff were overworked and oral care supplies were intermittently unavailable, critically ill patients may have suffered from reduced oral hygiene care. These factors may have contributed to an increase in plaque buildup in the severe group, rather than a predisposition to more serious disease.

This possibility of reverse causation significantly limits the interpretation of our results and emphasizes the need for future longitudinal studies in which oral hygiene status is assessed before or at the onset of infection. We explicitly caution against using our findings to advocate for oral hygiene interventions as a preventive strategy against severe COVID-19 without such temporal evidence.

In addition, we did not assess periodontal disease status beyond plaque accumulation, nor did we measure oral hygiene behaviors, smoking status, diabetes, obesity, cardiovascular disease, or vaccination status — all of which are established confounders in the relationship between oral health and COVID-19 severity. The lack of comprehensive medical records and the inability to conduct detailed interviews in overcrowded isolation centers prevented the collection of these critical variables. Without adjustment for these variables, the plaque index cannot be considered an independent determinant of disease severity. Future studies should include comprehensive periodontal examinations and multivariable regression analyses to clarify whether the observed association persists after controlling for these confounding factors.

### Critical evaluation of existing literature

The volume of literature examining the relationship between oral health and the severity of COVID-19 has grown significantly, yet the results are still mixed. While several studies reported positive associations between periodontal disease or poor oral hygiene and severe outcomes of COVID-19 [36, 39], other studies did not find a significant independent association after adjusting for confounders [41]. This discrepancy may be attributed to differences in study design (cross-sectional versus prospective), sample size, oral-health measurement methods (self-reported vs. clinical screening), degree of confounder control, and population characteristics.

The oral-lung axis hypothesis, while biologically plausible, is still not fully validated. Microaspiration of oral pathogens into the lower respiratory tract has been shown to be effective in mechanically ventilated patients, but the quantitative contribution of the oral biofilm to COVID-19 severity in particular has not been well established. Furthermore, the cytokine storm in severe COVID-19 cases is mainly driven by the virus-induced immune dysregulation, and the relative contribution of gingival inflammation to this process remains uncertain [42].

Importantly, the vast majority of published studies on this topic have been conducted in well-resourced healthcare settings with stable populations, comprehensive medical records, and advanced diagnostic capabilities. Our study contributes preliminary evidence from a highly resource-limited and conflict-affected context—an environment that is significantly underrepresented in the global scientific literature despite bearing a disproportionate burden of infectious diseases, morbidity, and mortality.

### Limitations

The present study has several important limitations. First, its cross-sectional design prevents conclusions about temporality or causation; therefore, we cannot determine whether higher plaque index preceded greater COVID-19 severity or whether severe illness itself impaired oral hygiene and increased plaque accumulation. In addition, the exact timing of plaque assessment in relation to oxygen therapy, non-invasive ventilation, ICU-level care, or other intensive supportive treatment was not consistently recorded, which may have influenced plaque accumulation during hospitalization.

Second, the sample size was small, with only 60 participants divided equally into three severity groups, which limited statistical precision, reduced generalizability, and prevented reliable multivariable regression analysis. The sample was based on consecutive eligible patients available in the participating COVID-19 centers during the study period, rather than population-based sampling.

Third, important potential confounders, including smoking status, diabetes mellitus, hypertension, obesity/BMI, nutritional status, medication use, vaccination status, socioeconomic status, oral hygiene behavior, and periodontal disease status, were not systematically collected and therefore could not be included in adjusted analyses. Therefore, plaque index should not be interpreted as an independent predictor of COVID-19 severity. These factors may be associated with both oral health and COVID-19 severity and may partly or fully explain the observed associations.

Fourth, the conflict-affected humanitarian setting may have introduced selection bias. The study was conducted in three COVID-19 isolation centers in northwest Syria during active conflict and healthcare disruption; therefore, patients treated at home, managed in peripheral clinics, or unable to reach these centers may have been underrepresented. Limited diagnostic capacity, restricted patient movement, and inability to perform longitudinal follow-up further reduced methodological strength. In addition, excluding patients who could not cooperate with oral examination because of severe respiratory distress or altered mental status may have introduced selection bias by underrepresenting the most critically ill patients. This may have led to underestimation or distortion of the association between plaque index and COVID-19 severity.

Fifth, plaque assessment was performed by a single trained examiner; although the use of a single examiner reduced inter-examiner variation, formal intra-examiner reliability testing was not performed, and measurement error cannot be excluded. In addition, because gingival index, bleeding on probing, periodontal pocket depth, and clinical attachment loss were not assessed, the study cannot determine whether the observed association reflects plaque accumulation, gingivitis, periodontitis, or broader oral inflammatory burden. Sixth, vaccination status was not systematically recorded, although vaccination is an important determinant of COVID-19 severity. Finally, the findings may not be generalizable to stable or well-resourced healthcare settings because the study population was affected by prolonged conflict, displacement, disrupted chronic disease care, limited preventive services, and shortages of healthcare resources. Thus, the results should be interpreted as preliminary evidence from a highly constrained humanitarian context.

## Conclusion

This study was conducted in northwest Syria during an active humanitarian crisis related to armed conflict, where healthcare infrastructure, diagnostic capacity, and patient follow-up were severely constrained. We observed statistically significant associations between COVID-19 symptom severity and male sex, older age, marital status, and dental plaque index. These findings provide preliminary evidence from an underrepresented population, but they should be interpreted cautiously because of the cross-sectional design, small sample size, and limited control of confounding variables.

Male sex was associated with greater COVID-19 severity, consistent with previous reports of worse outcomes among males [25, 26], possibly related to ACE2 expression and sex-based immune differences [27, 28]. Older age also showed a weak but significant positive association with severity, in agreement with published evidence identifying aging as an important risk factor for severe COVID-19 outcomes [30, 32, 33]. The association between marital status and severity should be interpreted cautiously because marital status was strongly related to age and may reflect confounding rather than an independent effect.

Higher dental plaque index was moderately associated with increased COVID-19 symptom severity. However, this finding does not imply causation. Because plaque index was measured after SARS-CoV-2 infection and during isolation or hospitalization, reverse causation remains possible; severe illness may have impaired oral hygiene and increased plaque accumulation, especially in a setting with limited nursing capacity and oral-care supplies. Therefore, plaque index should not be interpreted as an independent or modifiable risk factor for COVID-19 severity based on the present data. Because the study was not formally powered and included only 60 participants, the findings should be considered exploratory and hypothesis-generating rather than definitive.

Future longitudinal and multicenter studies are needed to clarify temporality, include comprehensive periodontal assessment, and adjust for key confounders such as smoking, comorbidities, obesity, vaccination status, socioeconomic status, oral hygiene behavior, and treatment-related variables. Until such evidence is available, oral health assessment may be considered as part of broader patient evaluation in resource-constrained settings, but not as an independent determinant of COVID-19 severity.

This study also highlights the value of reporting evidence from conflict-affected and humanitarian settings, where populations remain underrepresented in biomedical literature despite carrying a high burden of disease.

## Data Availability

Data availability: The datasets generated and analyzed during the current study are not publicly available due to patient confidentiality and institutional restrictions. De-identified data may be made available from the corresponding author upon reasonable request and after approval from the relevant institutional authority.
